# 

**Published:** 1893-11-04

**Authors:** 


**OUR NEW OFFICES,**
428, Strand, W.C., will henceforth be the Office of this Journal.

**Notices of Births, Deaths, and Marriages** are inserted in this column at a charge of 2s. 6d. for an announcement not exceeding four lines.

### NOTICE TO CORRESPONDENTS.

All MS., Letters, Books for Review, and other matters intended for the Editor should be addressed The Editor, The Lodge, Porchester Square, London, W.

The Editor cannot undertake to return rejected MS., even when accompanied by stamped directed envelope.

**Notice to Advertisers and Subscribers.**

All Advertisements, Orders for Copies of The Hospital, and Business Communications should be addressed to The Publisher, *not to the Editor*, The Hospital, 428, Strand, W.C.

The Cost of Subscription, post free, is as follows:—Per week, 2½d.; per quarter, 2s. 6d.; per half-year, 5s.; per annum, 8s. 6d. Foreign:—Per Annum, 10s. 8d.; payable, in all cases, in advance.

**"Burdett's Hospital Annual," 1893.**

Fourth year of publication. 700 pp. Boards, gilt lettering. A most complete guide to British, Colonial, Indian, and American Hospitals, Dispensaries, Nursing and Convalescent Institutions, and Asylums. Price 5s. Published by The Scientific Press (Limited), 428, Strand, W.C.

**NOTICE.—The Index for Vol. XIV. (ending Sept. 30th, 1893) is on sale at the Office of this Journal, price 2½d., post free.**

### SCRAPS AND GLEANINGS.

A PLEA has gone out at Peterhead for the erection of an infectious diseases hospital to meet the present outbreak of fever in the town.

A NEW ward with 30 beds has been added to the Seaside Convalescent Home, Seaford, Sussex. This home is one of the oldest of this class of institutions.

IT has been decided to hold a session of ambulance classes for both men and women at the Bolton Infirmary. Half-a-crown will be charged to each student.

EXTENSIVE alterations have been made to the Torbay Hospital at a total cost of about £8,962. It is hoped that the hospital will shortly be reopened, free of debt.

THE twenty-third annual report of the committee of the Newcastle Hospital Sunday Fund shows contributions to the amount of £4,525, being an increase of £194.

FIFTY brace of partridges have recently been sent to the Norfolk and Norwich Hospital by Mr. T. F. Mann, which has proved a most acceptable gift to the patients.

THE scheme of a charity football cup will be promoted by the Mayor of Leominster this winter in the interest of the cottage hospital which is proposed for that district.

THE Duke of Northumberland has forwarded £500 towards the building and extension fund of the Northern Counties Institute for the Blind at Newcastle. Earl Percy has contributed £50 towards the same object.

THE Marchioness of Abergavenny has given a harmonium to the East Sussex Hospital for the use of the new children's ward; in aid of this ward the matron has undertaken to arrange for a bazaar to be held shortly at St. Leonards.

THE first annual report of the committee of the Dumbarton Eye Dispensary is most satisfactory. During the year 938 patients were treated and 619 discharged cured. A balance of £20 remains in the treasurer's hands.

THE annual report of the committee of the Falmouth Dispensary shows a favourable balance-sheet. The committee find themselves unable to accept the conditional offer of £100 for the support of a children's ward in the new hospital.

THE Newbury Town and District Hospital Collections took place recently, when £132 was obtained. After paying expenses, £109 were given to the Newbury Hospital, £10 to the Newbury Dispensary, and the balance of £2 to the Cold Ash Children's Cottage Hospital.

THE accounts of the Medical Sickness, Annuity, and Life Assurance Society show that the society has attained a sound financial position. There is an increase of over 100 members during the past year, and the business shows steady signs of growth in all its branches.

THE annual Hospital Sunday Demonstration of the Friendly Societies of Park-street, Hertfordshire, took place recently. The offertories at all the services, and money collected in the streets, were divided between the St. Alban's Hospital and Hertfordshire Convalescent Home.

A MEETING was recently held at Bishop Stortford (Essex) to consider means of raising an annual income for a cottage hospital for the neighbourhood, towards which object £1,000 had been already promised by Mrs. and Miss Frere, in memory of the late Mr. Bartle J. L. Frere.

WE regret to hear that the Ottery St. Mary Cottage Hospital is in urgent need of increased support. Owing to unavoidable causes, the late Matron, who has generously provided the entire cost of maintenance, has had to discontinue her aid. An especial appeal has therefore been made.

THE Sanitary Committee of the Leeds City Council has adopted a report of the Hospital Sub-Committee with reference to the erection of a permanent hospital for infectious diseases on the Manston Hall Estate, which will involve an expenditure when fully carried out of about £100,000.

THE annual Hospital Demonstration and Church Parade of the Friendly Societies of St. Leonard's-on-Sea was recently held. After deducting expenses the proceeds were divided between the Friendly Societies' Convalescent Home (Dover), the St. Leonard's Convalescent Home, and the Tring Nursing Fund.

AT a meeting of the Sanitary Committee of Dundee Police Commission, convened to discuss the proposed small-pox and cholera hospital for the town, the tender of Mr. David Ogilvie, amounting to £229, was accepted. All vessels entering the river from cholera-infected parts will be detained until boarded by the medical officer.

THE work of the Birmingham Orthopædic Hospital has been successfully carried on during the past year. The financial condition of the charity, we are glad to state, has somewhat improved during the twelve months intervening since the last general meeting, the income for the year having exceeded the expenditure by £226.

THE amount of annual subscriptions already promised for the maintenance of the Ottery St. Mary Cottage Hospital (Exeter) is £217. Church offertories already secured amount to £25, but more have been promised. The total amount from all sources is now £270, and an additional £30 may be reckoned on from patients' fees.

THE Foresters of Salisbury recently held their annual Hospital Saturday and Sunday demonstrations. Throughout the preceding week collection boxes were placed in the hotels and restaurants, on the Saturday money was taken in the streets, while on the Sunday the church parade took place. The proceeds were given to the Salisbury Infirmary.

THE Earl of Leicester has presented £2,000 to the Norfolk and Norwich Hospital, in addition to a previous endowment of £15,000. Mr. W. Waring, of Taverham Hall, Norfolk, has transferred to the hospital £1,000 railway stock. The hospital has become a reversionary legatee to the extent of £1,000 under the will of the Rev. W. F. Thursby, deceased.

THE fever hospital on Freakes' Ground (Leicester) is to be utilised at last. The division between the small-pox and the fever wards will be made as complete as possible. The Sanitary Committee has at length determined to spend some hundred pounds on improving the existing accommodation in view of the widespread epidemic of scarlet fever in the locality.

A COMPLETE

### NURSE'S LIBRARY.

| | s. | d. |
|---|---|---|
| **THE THEORY AND PRACTICE OF NURSING:** By Percy G. Lewis, M.D. | 3 | 6 |
| **SURGICAL WARD-WORK AND NURSING:** By Alexander Miles, M.D. | 3 | 6 |
| **OPHTHALMIC NURSING:** By Sydney Stephenson, M.B. | 3 | 6 |
| **MENTAL NURSING:** By William Harding, M.B. | 2 | 6 |
| **THE NURSE'S DICTIONARY:** By Honnor Morten | 2 | 0 |
| **THE ART OF FEEDING THE INVALID:** By A Medical Practitioner | 3 | 6 |
| **A PRACTICAL HANDBOOK OF MIDWIFERY:** By Francis W. N. Haultain, M.D. | 5 | 0 |
| **THE ART OF MASSAGE:** By A. Creighton Hale | 6 | 0 |
| **HOSPITAL SISTERS AND THEIR DUTIES.** By Eva C. E. Lückes | 2 | 6 |
| **HOW TO BECOME A NURSE:** By Honnor Morten | 2 | 6 |
| **THE NURSE'S CASE-BOOK:** For Hospital and Private Uses | 0 | 6 |

The Complete Set, Carriage Paid, £1 10s.

CATALOGUES SENT POST FREE TO APPLICANTS.

London: The Scientific Press, Limited, 428, Strand, W.C.

### Medical Vacancies and Appointments.

### APPOINTMENTS.

J. Adams, M.D.St.And., M.R.C.S.Eng., appointed Medical Officer of Health for the Barnes Urban Sanitary District. G. F. Aldous, L.R.C.P.Lond., M.R.C.S.Eng., reappointed Medical Officer to the Provident Department of the Plymouth Public Dispensary. F. Borough, M.R.C.S.Eng., reappointed Surgeon to the Derby Provident Dispensary. W. F. Brook, F.R.C.S.Eng., L.S.A., appointed Visiting Surgeon to the Swansea General Hospital. W. A. Buchan, M.B., C.M.Edin., reappointed Medical Officer to the Provident Department of the Plymouth Dispensary. Butler-Hogan, B.A.R.U.I., M.D.Brux., L.R.C.P., L.R.C.S.Edin., D.P.H.Cantab., appointed Medical Officer of Health for Shoreditch. J. Caithness, M.D., C.M.Glasg., reappointed Medical Officer for the Llanrhaiadr District of the Ruthin Union. W. G. Copestake, M.R.C.S.Eng., reappointed Surgeon to the Derby Provident Dispensary. W. J. Corrigan, L.R.C.S.I., L. & L.M.K.Q.C.P.I, appointed Surgeon to the Dowlais Ironworks, Cardiff. W. G. Curgenven, M.D.St. And., M.R.C.S.Eng., reappointed Surgeon to the Derby Provident Dispensary. F. J. H. Coutts, M.B., Ch.B.Vict., appointed Resident Medical Officer to the Victoria Hospital, Burnley. J. Davies, L.R.C.P., L.R.C.S.Edin., appointed Honorary District Medical Officer to the Ladies' Charity and Lying-in Hospital, Liverpool. J. L. Dick, M.B., C.M.Edin., appointed House-Surgeon to the Clinical Hospital for Women and Children, Manchester. C. J. W. Dixon, M.B., C.M.Edin., appointed Resident Medical Officer at the Royal Hospital for Diseases of the Chest, City Road. A. Eddowes, M.D., M.R.C.S., appointed Physician to the Western Skin Hospital. H. J. Foulds, M.R.C.S.Eng. reappointed Surgeon to the Derby Provident Dispensary. D. A. Fraser, M.D., M.R.C.S., L.S.A., reappointed Medical Officer of Health for Totnes Borough. W. A. Frost, F.R.C.S., appointed Ophthalmic Surgeon to St. George's Hospital. J. Little, M.D.Edin., M.R.C.S.Eng., appointed Medical Officer of Health to the Maryport Urban Sanitary District. L. S. Luckham, M.R.C.S.Eng., L.S.A., appointed Medical Officer of the Workhouse of the Alderbury Union. J. Orr, M.B., C.M.Edin., L.R.C.P., M.R.C.S.Eng., appointed Resident Medical Officer to the Chalmers Hospital, Edinburgh. C. F. Pridham, B.A.Cantab., M.R.C.S., L.R.C.P.Lond., appointed Medical Officer of the Tenth District of the Wycombe Union. A. H. Rees, L.R.C.P. Edin., M.R.C.S.Eng., reappointed Assistant Physician to the Plymouth Public Dispensary. H. W. Scratchley, F.R.C.S., L.R.C.P., reappointed Medical Officer for the Third District of the Poole Union. A. J. Southey, M.R.C.S.Eng., L.S.A., reappointed Medical Officer of Health to the Slough Urban Sanitary District. W. R. Smith, M.B.Lond., M.R.C.S., L.R.C.P., L.S.A., appointed House Surgeon to the St. Peter's Hospital for Stone, Henrietta Street, Covent Garden. F. E. Taylor, M.R.C.S. Eng., reappointed Surgeon to the Derby Provident Dispensary. E. Vaudry, M.D.Edin., M.R.C.S.Eng., reappointed Surgeon to the Derby Provident Dispensary. D. Walsh, M.B., C.M.Edin., appointed Assistant Physician to the Western Skin Hospital, Great Portland Street, W. J. E. C. Wilson, L.R.C.P., L.R.C.S. Edin., reappointed Assistant Physician to the Plymouth Public Dispensary. J. L. Wright, L.R.C.P.Edin., M.R.C.S.Eng., reappointed Surgeon to the Derby Provident Dispensary.

### VACANCIES.

The following vacancies are announced this week, the amount of salary in each case being given after the name of the institution:

**Resident Medical Officers.**

Cheltenham General Hospital. Resident Surgeon for the Branch Dispensary; unmarried, or if married, without family. Salary £180 per annum, with partly-furnished house, coal, and gas. Applications to the Honorary Secretary and Treasurer by November 25th.

Hospital for Sick Children, Great Ormond Street, Bloomsbury, W.C. Resident Medical Officer as House Physician; unmarried. Appointment for six months. Salary £20, with board and residence in the hospital. Applications to Secretary by November 7th.

National Sanatorium for Consumption and Diseases of the Chest, Bournemouth. Resident Medical Officer and Secretary. Salary £120 per annum, with board and lodging. Applications to the "Chairman of Committee" by December 5th.

Royal National Hospital for Consumption, Ventnor. Assistant Resident Medical Officer; unmarried. Salary £70 per annum, with board and lodging in the hospital. Applications to be addressed to the Board of Management at the London office, 34, Craven Street, Charing Cross, W.C.

Torbay Hospital and Provident Dispensary, Torquay. Junior House Surgeon and Dispenser; doubly qualified, unmarried. Board, lodging, and attendance provided. Salary £80 per annum. Applications to the Honorary Secretary, Captain Phillpotts, R.N., by November 13th.

**Non-Resident Medical Officers.**

Belgrave Hospital for Children, 77 and 79, Gloucester Street, S.W. Physician for Outpatients. Applications to Honorary Secretary by November 8th.

Birmingham and Midland Free Hospital for Sick Children. An Acting Physician and a Surgeon Dentist. Appointment for twelve months, but eligible for re-election. Applications to the Medical Board, Children's Hospital, Steelhouse Lane, Birmingham, by November 8th.

Hospital for Women, Soho Square, W. Assistant Physician. Applications to the Secretary by November 14th.

### NOTES FROM THE SCHOOLS.

**Guthrie Society, Westminster Hospital.**

The inaugural smoking concert of the society for the session 1893-94 was held at the Medical School at half-past eight p.m. The newly-elected President, Dr. Donkin, was in the chair, and was supported by the following members of the staff: Dr. Sturges, Dr. Hebb, Mr. Bond, Mr. Black, Dr. Marett Tims, and Mr. De Santi. Fifty members were present, and seven visitors. Twelve new members were elected. A most successful smoking concert was then proceeded with, in which the visitors rendered much appreciated assistance. Toward the end of the evening Dr. Sturges rose to congratulate the President on the good attendance. He referred to the many advantages there were for the students at the hospital medical school, and especially for those who were members of the Guthrie Society. Mr. Bond, in a short speech, which was much appreciated, seconded the vote of congratulation to the President. The President replied. The programme for the evening was then continued, only a very few of the members retiring before half-past eleven, when the President announced that at the next meeting, November 9th, Mr. E. Sherlock, B.Sc.Lond., would read a paper entitled "The Theory of Recapitulation," to be followed by "My Experiences as a Railway Surgeon," by Mr. Thomas Bond, F.R.C.S., London.

**London Hospital Medical College.**

The complete list of entrance scholarships at the London Hospital Medical College are as follows: First Entrance Science, value £75, Mr. C. W. Chaplin; Second Entrance Science, value £50, divided equally between Mr. H. G. Frankling and Mr. Alfred Orme; First Buxton Scholarship (Arts), value £30, Mr. F. M. Morris; Second Buxton Scholarship, value £20, Mr. J. F. Walker.

**St. Mary's Hospital Medical School, Paddington.**

The scholarships for the winter session 1893-94 have been awarded as follows: University Scholarships of £52 10s. to W. Fletcher, B.A.Cantab., and H. H. Knapp, B.A.Oxon.; Epsom Scholarship of £135 to S. M. Smith; Natural Science Scholarships of £52 10s. each to F. S. Dawe, R. C. Leaning, and A. E. Horn.

**St. George's Hospital Medical School.**

The following Entrance Scholarships have been awarded: An Entrance Scholarship in Arts of £145, for sons of medical men, to Mr. H. S. Barwell; University Entrance Scholarships of £85, in Anatomy and Physiology to Mr. F. J. Watson, of Trinity College, Cambridge, and to Mr. Arthur C. Latham, of Balliol College, Oxford. An Entrance Scholarship in Arts of £50 to Mr. R. E. Drake-Brockman.

### PASS LISTS.

**University of Oxford.**

An examination will be held on November 21st for Natural Science scholarships at Balliol and Trinity Colleges and at Christ Church. Four scholarships of £80 per annum and three exhibitions (value from £85 to £40 per annum) will be awarded. Papers will be set in (1) Physics, (2) Chemistry, and (3) Biology, but candidates will not be expected to offer more than two of these. Candidates must communicate with the Master of Balliol by letter before November 14th.

**University of Edinburgh.**

The following gentlemen have passed the final examination for the degrees of M.B. and C.M.: F. O. Bell, W. Carr, W. B. Clarke, E. A. Dent, R. A. Dove, P. G. Edgar, C. R. Edmondson, W. Forsyth, W. H. O. Gaunt, H. F. Green, J. K. D. Ingrom, O. H. Isard, D. S. Johnston, C. W. A. Jones, E. Jones, J. G. Leslie, A. H. Lowe, D. Macdonald, A. G. M'Intyre, H. D. N. Mackenzie, J. Michael, T. P. Monteath, B.A., J. L. I. Moore, R. D. O'Neale, J. L. Owen, F. S. Park, J. H. Phyn, A. F. M. Powell, M. M. Rattray, E. A. M. Roberts, H. A. Robertson, A. H. Robinson, A. A. Ross, G. C. Sandford, A. C. Smith, R. Stephens, R. Tandan, W. A. Taylor, T. J. Thomson, J. A. Thorne, A. C. Turner, R. B. Wallace, G. Warnes, and H. Weighton.

### The Hospital Advertiser.

### MEDICAL APPOINTMENTS.

**TORBAY HOSPITAL AND PROVIDENT DISPENSARY, TORQUAY.**

